# Ventilator-Induced Lung Liquid and Alveolar Rupture

**DOI:** 10.3390/jcm15134884

**Published:** 2026-06-23

**Authors:** Jesús Villar, Stephen M. Pastores

**Affiliations:** 1 CIBER de Enfermedades Respiratorias, Instituto de Salud Carlos III, 28029 Madrid, Spain; 2 Research Unit at Hospital Dr. Negrín, Fundación Canaria Instituto de Investigación Sanitaria de Canarias, 35019 Las Palmas de Gran Canaria, Spain; 3 Li Ka Shing Knowledge Institute, Sta Michael Hospital, Toronto, ON M5B 1W8, Canada; 4 Faculty of Health Sciences, Universidad del Atlántico Medio, Tafira Baja, 35017 Las Palmas de Gran Canaria, Spain; 5 Critical Care Center, Department of Anesthesiology and Critical Care Medicine, Memorial Sloan Kettering Cancer Center, 1275 York Avenue, New York, NY 10065, USA; 6 Medicine in Clinical Anesthesiology and Medicine, Weill Cornell Medicine, New York, NY 10065, USA

**Keywords:** ventilator-induced lung liquid and alveolar rupture, mechanical ventilation, lung protection, fluid management, baby-lung, driving pressures, outcomes

## Abstract

Ventilation is an essential function of life, and one of the first to be replicated by artificial means. Annually, it is estimated that 15 to 20 million patients worldwide are intubated and receive invasive mechanical ventilation (MV). However, MV is a non-physiologic intervention and frequent complications are associated with its use, including extravascular lung liquid, impaired cardiac performance, and alveolar rupture. Research shows that injurious MV can cause or aggravate lung damage and initiate an intense inflammatory response, contributing to multiple organ dysfunction and poor outcomes due to ventilator-induced lung liquid and intense alveolar rupture. In this brief commentary, we postulate that this resulting injury is better characterized with the term “ventilator-induced lung liquid and alveolar rupture”. We will summarize key points for clinical implications, existing challenges, and future perspectives for the management of patients with severe acute hypoxemic respiratory failure.

## 1. Introduction

**Figure 1:**
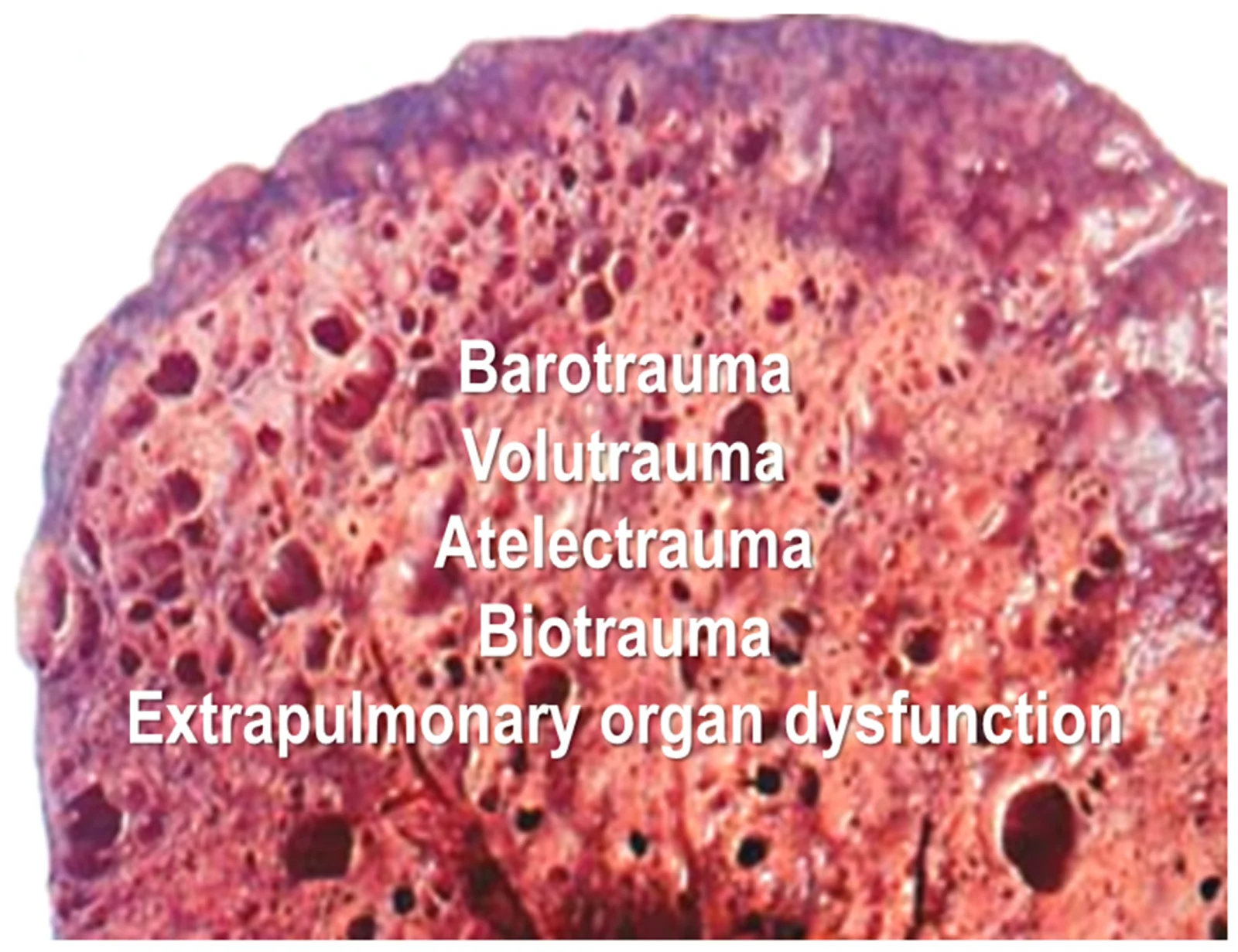
The five components of ventilator-associated lung liquid and alveolar rupture. The background represents a grossly fibrotic appearance of a lung from a patient with acute respiratory distress syndrome caused by recurrent aspiration who died in the early 1980 after a few weeks of mechanical ventilation at high tidal volumes.

Ventilation is an essential function of life, and one of the first to be artificially replicated. An estimated 15 to 20 million patients receive mechanical ventilation (MV) annually worldwide [1]. It is a frequently used intervention in intensive care units (ICUs). Despite its necessity, MV is a non-physiologic intervention, and its use carries significant risks, including barotrauma, impaired cardiac performance, and increased alveolar flooding [2,3]. Injurious MV can cause or aggravate the damage to the lungs and initiate a systemic inflammatory response, contributing to multiple system organ dysfunction and poor clinical outcomes [3,4]. Several specific forms of ventilator-induced lung injury have been identified including alveolar wall rupture (barotrauma), overdistension or over-stretching of alveoli due to excessive gas volume (volutrauma), cyclic atelectasis or repeated alveolar collapse and expansion in the lung caused by MV (atelectrauma), overstretching or shearing during MV induced by biomarker release (biotrauma), and extrapulmonary organ trauma, all leading to poor clinical outcomes [2,3,4,5,6] (as shown in Figure 1).

During MV, airway pressures, inspiratory gas volumes, pressure or respiratory rates, and oxygen concentrations are applied far exceeding levels that normal lungs usually experience. We define this resulting injury as ventilator-induced lung liquid and alveolar rupture (VILLAR), and it closely resembles the acute respiratory distress syndrome (ARDS). VILLAR is characterized by alveolar-capillary membrane disruption, leading to increased permeability, interstitial and alveolar lung liquid, and alveolar rupture as a direct consequence of intense or injurious application of MV. Although patients may be demographically and physiologically comparable, various types of lung injury can yield a different prognosis despite a similar pulmonary response. VILLAR is a discrete entity; as a result, it is difficult to identify in humans because its presentation typically overlaps with the underlying disease process. In VILLAR, the flooding of air spaces by transudate, exudate, or pus results from mechanical disturbances to the alveolar-capillary membrane. This lung inflammation is accompanied by many cellular and biochemical processes: some may initiate the VILLAR condition, others may perpetuate the injury, and still others might attempt to mitigate the inflammatory sequelae. Consequently, for nearly three decades, a paradigm shift in MV has prioritized lower tidal volumes and accepting ‘permissive’ gas-exchange values to mitigate further injury. There is no typical ARDS patient. Since no specific clinical sign or diagnostic test has yet been described that identifies ARDS, its diagnosis is based on a constellation of clinical, chest imaging, and oxygenation criteria.

In the words of Sir William Osler, “Variability is the law of life and as no two faces are alike, no two individuals react alike and behave alike under the abnormal condition we know as disease” [7]. The term evidence-based medicine is just a verdict based on science that the proposed intervention has the highest probability of success: We seek the truth, but how honest is the literature on which we base our verdict? for a particular disease process. Since medicine is a science of uncertainty and an art of probability, the summit of evidence-based medicine is the randomized controlled trial (RCT).

The recognition of VILLAR has led us to suggest that ARDS, the most severe form of acute hypoxemic respiratory failure in critically ill patients, may be, in part, a product of our ventilatory interventions rather than mere progression of the underlying disease. ARDS patients provide clinicians and researchers with complicated and multifactorial problems. No single mode of ventilation is universally applicable. Despite many RCTs and established guidelines advocating for changes in clinical practices, a significant “evidence-practice gap” persists, as many clinicians remain tethered to traditional practices [8,9]. Despite advancements, the optimal approach to MV remains a subject of ongoing research [3].

This brief commentary examines the evidence supporting the concept of VILLAR, its clinical implications, current challenges in its identification, and future perspectives for the personalized management or tailoring of therapeutic strategies to individual immunological profiles of patients with ARDS [5].

## 2. VILLAR Concept

VILLAR involves injury to both the capillary endothelium and the alveolar epithelium, although lung dysfunction is always characterized by injury to both barriers. In general, it is useful to think of the pathophysiological mechanisms as a result of two different pathways: direct lung cellular insult and the indirect result of an acute systemic inflammatory response. In VILLAR, the presence of lung liquid under high mechanical pressures acts as a physical force that causes “liquid-induced” rupture of the alveolar walls, leading to more rapid and severe structural failure than air-stretch alone. The mechanical force of the ventilator, applied to a lung that already contains liquid, creates a “liquid-solid” interaction that physically breaks the lung structure. This physical “break” can occur even before the full biological inflammatory cascade of injury has fully set in. Management of VILLAR requires a targeted, multi-modal strategy, including active negative fluid balance, which could be useful to reduce lung edema and administration of corticosteroids to mitigate lung inflammation due to the main cause of ARDS or from injurious MV. The justification for active negative fluid balance and/or administration of corticosteroids includes: (i) reduced extravascular lung water, (ii) optimal fluid status, (iii) improved short-term and long-term outcomes; (iv) prevention of “third space”; (v) anti-inflammatory and anti-fibrotic impact; and (vi) reduced days on MV and associated length of ICU stay. As suggested in a very recent narrative review [10], early positive fluid balance is linked to longer intensive care unit (ICU) stays, prolonged ventilatory support, and increased mortality risk due to cardiopulmonary complications, lung edema, and extrapulmonary organ dysfunction. The 2006 ARDS Network Fluid and Catheter Treatment Trial (FACTT) [11] established that while neither a conservative nor liberal fluid strategy affected the primary outcome of 60-day mortality, the conservative approach improved lung function and shortened the duration of MV and intensive care without increasing nonpulmonary organ failures. These results support the use of a conservative strategy of fluid management in patients with ARDS.

Currently, we lack sufficient knowledge to draw definitive conclusions regarding the precise sequence of events that determine which of these putative pathophysiological mechanisms are most critical in the development of VILLAR. Uncovering the mechanisms responsible for VILLAR is the most important obstacle to the successful treatment of patients with severe ARDS. It is increasingly evident that mortality of ARDS is unlikely to decrease below 30% until we develop and institute specific, precision-based therapies early enough to prevent the underlying inflammatory state and subsequent multiple system organ dysfunction. Given the phenotypic heterogeneity observed across numerous RCTs and observational studies in ARDS, the identification of putative management with optimal driving pressure [12] and optimal positive end-expiratory pressure (PEEP), timing for prone positioning, or “true” subphenotyping of critically ill patients that could change outcome is difficult [13]. It is plausible that stratification of respiratory and ventilatory variables could help identify and select patients for targeted RCTs.

## 3. Clinical Implications, Existing Challenges, and Future Perspectives

Managing lung tissue represents one of the most challenging problems in critical care medicine, despite advances in terms of lung mechanics and physiology, prevention of any cause of ARDS, management of multiple organ dysfunction, machine learning models, and appropriate application of artificial intelligence for diagnosis and treatment [3]. To effectively practice modern critical care medicine, clinicians must be knowledgeable of the complete spectrum of clinical disease and the safety and efficacy of all treatments for any disease. The better we understand the quality of the evidence we use to make clinical decisions, the better we can judge whether new scientific evidence should be incorporated into our practice. Patients with VILLAR are often ventilated with tidal volumes derived from estimates of body size despite having greater than 50% of air space flooding. If the ARDS lung is modeled in two regions, one nearly normal and having dimensions similar to those of a healthy baby [14]. The restricted capacity of this “baby lung” is responsible for the mechanical characteristics described I ARDS [15]. While studies of VILLAR represent the study of inflammation in the lung, these effects may differ in some respects from inflammation elsewhere, although it is likely they involve several of the same mechanisms. In the early 1960s, anesthesiologists and critical care physicians showed that small-volume tidal ventilation caused a gradual loss of lung volume and hypoxemia due to right-to-left shunting through regions with poor ventilation. As a result, the one-tidal volume fits all approach was formulated, and inspiratory volumes of 10–15 mL/kg body weight were recommended until the year 2000 [16]. However, experts and pioneers in critical care medicine continued ventilating patients with ARDS with large tidal volumes and very high pressures. The amount and quality of information regarding MV in the injured lung is much greater now than in the 1960s. As a general pattern, all mammals are biologically scaled similarly to a tidal volume of approximately 6.3 mL/kg body weight [6]. Although we cannot definitively say which tidal volume, inspiratory or expiratory pressures, or ventilatory rates have more impact on VILLAR outcome, we can say without reservation that the era of large tidal volumes or high inspiratory pressures must end. VILLAR also stresses the importance of managing the “liquid” component and transpulmonary pressures specifically to prevent the physical rupture of the alveoli caused by liquid movement.

The mechanical overinflation of lung tissue causes overactivation of the immune system. In experimental models, healthy lungs ventilated with large tidal volumes evoke early inflammatory responses similar to those induced by endotoxin. The combination of high tidal volumes and zero PEEP exerts a synergistic effect on cytokine gene expression in pre-injured lungs [17]. Since patients with ARDS are monitored extensively, the main goal of MV is to reverse and prevent hypoxemia, reduce excessive work of breathing, prevent further injury, and track the progression of lung repair. Driving pressures (calculated as the difference in plateau pressure minus PEEP) should be maintained as low as possible, strictly below 15 cm H_2_O [12], and plateau pressures should be less than 30 cm H_2_O. Consequently, tidal volumes should generally be titrated between 4 and 8 mL/kg predicted body weight. However, personalized MV strategies will only succeed if we avoid the misclassification of ARDS subphenotypes. To prevent repeated alveolar collapse and re-expansion, PEEP should be applied to ensure that the recruited lung is maintained open, typically within the 8 to 12 cm H_2_O range. Electric impedance tomography shows potential promise as a bedside monitoring tool for real-time visualization of lung volumes and regional ventilation distribution. The future of MV is shifting towards these personalized, lung-protective strategies. Mechanical power has emerged as a unifying metric to quantify the intensity of the physical effects of MV applied to the lung parenchyma. By integrating the various drivers of lung injury into the ventilator software expressed as energy over time (J/min), we can technologically monitor the cumulative risk of VILLAR in real-time [18].

The identification of interventions that change outcomes remains extremely difficult. Outcome reporting in RCTs and observational studies of ARDS is highly heterogeneous, highlighting the urgent need for a standardized, patient-centered core outcome set to improve comparability across trials and cohorts of ARDS. It is a fallacy to believe that any randomized group is truly ‘well matched’; intra-group heterogeneity is an indisputable reality, and applying aggregate trial results to an individual patient can, in some instances, be detrimental. Therefore, mathematical significance and clinical relevance are not synonymous. For example, ARDS RCTs exhibit substantial variability in baseline characteristics and a striking heterogeneity in 28-day control group mortality [19]. There are three major reasons to explain this ‘evidence-practice’ gap: (i) patients enrolled in RCTs are frequently not representative of the standard ICU population. (ii) the outcome under study (mortality in most) may be relevant but not in the way reported. For example, despite that sepsis or pneumonia are one of the most frequent diagnoses in the ICU (claimed by many authors), many sites contribute very few patients to large sepsis or pneumonia RCTs, which calls into question the generalizability of their findings; and (iii) reporting and supporting the evidence does not mean that medical practice should change solely on the findings of one positive RCT. If the subjects of a trial have a very low risk of the condition that the intervention is hypothesized to prevent, the trial (regardless of the sample size) will not prove the value of the intervention [20]. Because nearly all ARDS patients present with severe oxygenation deficits (measured with PaO_2_/FiO_2_ or SpO_2_/FiO_2_ ratios), there is little room for meaningful stratification unless hypoxemia is evaluated under standardized ventilatory settings. This illustrates the major problems in trying to compare the efficacy of various clinical trials of MV strategies.

Sepsis and pneumonia remain the major causes of ARDS, often suppressing multiple system organ functions, and serving as the leading causes of global morbidity and mortality. Future trials when treating sub-phenotypes as treatable traits, or validating prone positioning and comparing corticosteroids to a non-corticosteroid control in severe ARDS, may be challenging due to the unwillingness among centers to randomize patients to receive no anti-inflammatory mediators, no prone position, or no corticosteroids in pneumonia or sepsis. Furthermore, the outcome differences between personalized MV subphenotypes (depending on the morphology of lung injuries, mortality predictors, lung ultrasound findings, and etiology) complicate the identification of universal treatable traits. We need to validate the PROSEVA trial on prone positioning since it reported the lowest recorded 28-day mortality rate in severe ARDS) (16% vs. 32.8% in the supine group) [21]. The physiological benefits of prone positioning are well documented, including: (i) improved ventilation from augmented functional residual capacity, (ii) favorable changes in regional diaphragmatic excursion, (iii) better secretion removal, and (iv) redistribution of perfusion away from previously dependent and more edematous lung regions.

Future research must individualize corticosteroid dosing based on the specific alveolar immuno-inflammatory status of the patient. As highlighted in a recent narrative review [22], contemporary trials and meta-analyses indicate that early administration of systemic corticosteroids at receptor-saturating doses significantly accelerates the resolution of pulmonary edema, shortens the duration of MV, and improves overall ICU survival.

Treatment of ARDS should be individualized. Factors like hemodynamic status, kidney function, timing, and the phase of fluid removal are important for personalized medicine [9]. Notably, prognostic equivalence does not imply therapeutic equivalence. We have to develop statistical multivariable adjustment and machine learning (ML) models for associating MV modalities and ICU mortality. Once a patient is discharged from the ICU, the intensivist’s direct control over patient care ceases. While it is questionable whether post-ICU hospital outcomes are directly related to ICU ventilator management, combining ICU and hospital mortality rates remains vital for interpreting RCT findings. Ultimately, artificial intelligence (AI) and ML algorithms are not intended to replace the ICU physician’s clinical judgment. Instead, their true value lies in the near future ability to synthesize the complex data structures generated by the entire care team (physicians, nurses, respiratory therapists, and pharmacists) working with the same critically ill patient [23].

The Berlin ARDS definition remains the only validated criteria, recognized by the European Society of Intensive Care Medicine and other major professional societies [24]. The most recent extensions of the ARDS definition [13] have sought to increase diagnostic sensitivity at the cost of specificity. By expanding the clinical criteria, these updates continue to complicate the identification of patient subgroups at imminent risk of death or those who might benefit from therapies targeting specific pathogenic mechanisms. Furthermore, while the “global” definition includes the Kigali modification [25], it lacks universal recognition. Given that the core tenets of the ARDS definition were established nearly 15 years ago, a more precise, contemporary definition is needed to better characterize human ARDS, supported by a frequently updated implementation process.

We need to introduce measurements of hypoxemia under standardized ventilatory settings and identify risk factors that classify pulmonary and systemic severity. Specific biomarkers of VILLAR for “true” subphenotypes of patients should be part of the new definition [26]. Ideally, such biomarkers should be: 100% sensitive, 100% specific, easy to measure in blood, exhaled air, or other biological samples, responsive to treatment and capable of predicting survival, and cost-effective. If ARDS outcomes are inextricably linked to ventilatory settings, our patients require that we proceed with the translational and personalized application of MV research without undue skepticism. Additional research on VILLAR is needed. It is also plausible that a new definition based on specific biomarkers or biochemical criteria of lung inflammation, rather than on clinical parameters, will provide us with a more homogeneous patient population. This stratification of ARDS patients should be linked to two measures of severity: one that specifically quantifies the verity of ARDS, and another that quantifies the patient’s overall physiologic response along with comorbidities and premorbid conditions. It is plausible to think that predictive models using AI and ML algorithms could be developed to identify VILLAR-susceptible patients [3].

In summary, we cannot yet definitively identify which individualized tidal volume or inspiratory/end-expiratory pressure and fluid management strategy has the most impact on clinical outcomes. However, the recognition of VILLAR as a discrete, preventable injury provides a new framework for both clinical practice and academic inquiry. Rigorous investigations and close collaboration between intensivists, pulmonologists, anesthesiologists, nurses, and researchers, to prioritize and sustain high-quality RCTs and minimize research waste, is essential to treat this catastrophic condition and improve the survival of ventilated patients.

## Data Availability

Not applicable.
